# From gene to mechanics: a comprehensive insight into the mechanobiology of *LMNA* mutations in cardiomyopathy

**DOI:** 10.1186/s12964-024-01546-5

**Published:** 2024-03-27

**Authors:** R. J. A. Veltrop, M. M. Kukk, K. Topouzidou, L. Didden, A. Muchir, F. G. van Steenbeek, L. J. Schurgers, M. Harakalova

**Affiliations:** 1 Department of Biochemistry, CARIM, Maastricht University, Maastricht, The Netherlands; 2 Department of Cardiology, Division Heart and Lungs, University Medical Center Utrecht, Utrecht, the Netherlands; 3 Regenerative Medicine Utrecht, University Medical Center Utrecht, Utrecht, The Netherlands; 4 LMNAcardiac.org, LMNA Patient Organization, Soest, The Netherlands; 5 Striated Muscle Laminopathy Consortium, ENMC, Baarn, The Netherlands; 6 Centre de Recherche en Myologie, U974 SU-INSERM, 75013 Paris, France; 7 Department of Clinical Sciences of Companion Animals, Faculty of Veterinary Medicine, Utrecht University, Utrecht, The Netherlands

**Keywords:** Cardiolaminopathy, Mechanotransduction pathways, Kinase activity, Dynamic reciprocity, Mechanobiochemistry

## Abstract

Severe cardiac remodeling leading to heart failure in individuals harboring pathogenic LMNA variants, known as cardiolaminopathy, poses a significant clinical challenge. Currently, there is no effective treatment for lamin-related diseases. Exploring the intricate molecular landscape underlying this condition, with a specific focus on abnormal mechanotransduction, will propel our understanding of cardiolaminopathy. The *LMNA* gene undergoes alternative splicing to create A-type lamins, a part of the intermediate filament protein family. A-type lamins are located underneath the nuclear envelope, and given their direct interaction with chromatin, they serve as mechanosensory of the cell by interacting with the cytoskeleton and safeguarding the transcriptional program of cells. Nucleated cells in the cardiovascular system depend on precise mechanical cues for proper function and adaptation to stress. Mechanosensitive signaling pathways are essential in regulating mechanotransduction. They play a pivotal role in various molecular and cellular processes and commence numerous downstream effects, leading to transcriptional activation of target genes involved in proliferation, migration, and (anti-)apoptosis. Most pathways are known to be regulated by kinases, and this area remains largely understudied in cardiomyopathies.

Heart failure is linked to disrupted mechanotransduction, where *LMNA* mutations affect nuclear integrity, impacting the response to extracellular matrix signals and the environment. The Hippo pathway, anchored by YAP1/WWTR1, emerges as a central player by orchestrating cellular responses to mechanical signals. However, the involvement of Hippo and YAP1/WWTR1 in cardiolaminopathy is unclear and likely mutation- and tissue-specific, warranting further investigation. Here, we highlight the involvement of multiple signaling pathways in mechanotransduction in cardiolaminopathy. We delve into (non-)canonical functions of key signaling components, which may hold critical clues for understanding disease pathogenesis. In summary, we comprehensively examine the mechanobiology of A-type lamins, the role of mechanosensitive signaling pathways, and their intricate interplay in the pathogenesis of cardiolaminopathy. A better understanding of these mechanisms is paramount for developing targeted therapies and interventions for individuals afflicted with this debilitating cardiac condition. Prior studies overlooked accurate gene nomenclature in protein and pathway names. Our review addresses this gap, ensuring precision by aligning names with correct gene nomenclature.

**Supplementary Information:**

The online version contains supplementary material available at 10.1186/s12964-024-01546-5.

## Abstract (plain language summary)

Mutations in the A-type lamin gene (*LMNA*) can cause a laminopathy. A specific manifestation of this disease leads to cardiolaminopathy, a serious heart condition. The lamin network, located at the inner nuclear membrane, is a central player in transforming forces within cells. As cells move and function, they rely on the ability to sense and respond to these forces, a process named mechanosensing and -response. This review provides an overview of the key molecular pathways involved in the development of heart failure. The molecular mechanisms underlying *LMNA* cardiomyopathy are poorly understood because the interaction between the signaling pathways is challenging to elucidate. Deciphering these pathways is key to understanding the underlying mechanisms of disease and finding novel targets to alter the pathways and lessen the symptoms of diseases.

**Supplementary Information:**

The online version contains supplementary material available at 10.1186/s12964-024-01546-5.

## Background

**Fig. 1:**
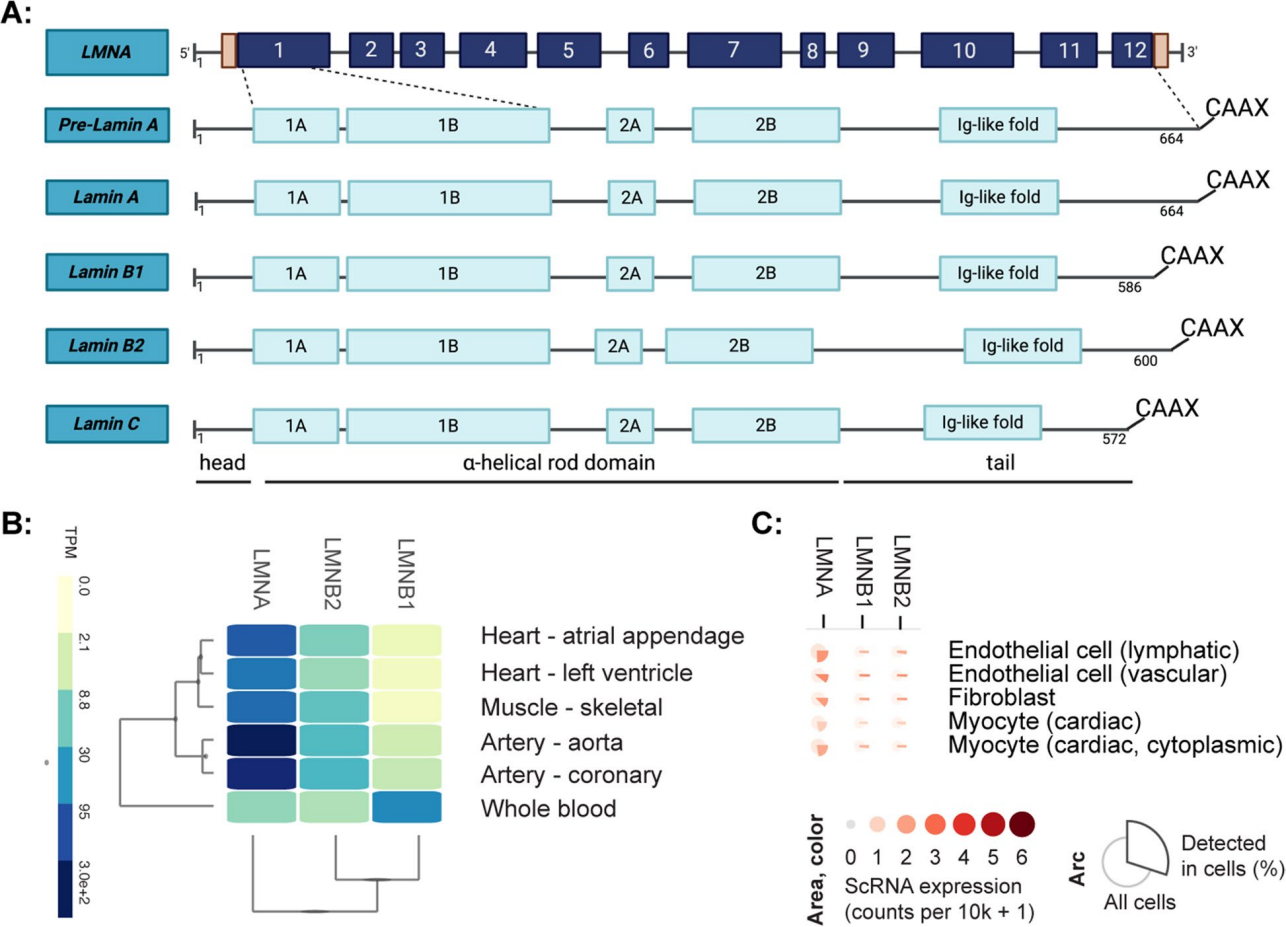
Graphical representation of the lamin isoforms. **A** Lamin A, B and C are isoforms resulting from alternative splicing and post-translational processing of the primary transcript of the LMNA and LMNB genes. **B** Bulk RNA-seq data of LMNA and LMNB in the cardiovascular system. **C** Single-cell RNA expression profiles of LMNA and LMNB in selected cell types within the left ventricle of the heart (source: Multi-Gene Single Cell Viewer from GTEx)

The discovery of an association between mutant A-type lamins and disease dates back to 1999 when Bonne and co-workers uncovered mutant *LMNA* in an autosomal dominant form of Emery-Dreyfuss muscular dystrophy (EDMD) [1]. Additionally, Fatkin et al. reported a link between *LMNA* and dilated cardiomyopathy (DCM) [2]. A-type lamins are type V intermediate filament (IF) proteins located on the underside of the inner nuclear membrane [3, 4], where they polymerize to form a nuclear lamina meshwork interacting with B-type lamins. A-type lamins are also found in the nucleoplasm [5]. Like other type V IF proteins, lamins consist of a globular head and tail domain with an immunoglobulin-like fold, separated by a helical rod domain [6]. Lamins can broadly be categorized into A-type and B-type lamins. Although this review primarily focuses on A-type lamins, it is important to understand their differences. B-type lamins are encoded by *LMNB1* and *LMNB2* genes. Lamin A and lamin C are collectively referred to as A-type lamins and are isoforms resulting from alternative splicing and post-translational processing of the primary transcript of the *LMNA* gene [7]. Lamin A and C are identical until the 10th exon – while lamin A messenger ribonucleic acid (mRNA) contains 12 exons, lamin C lacks the last two exons [8]. Lamin A undergoes farnesylation (post-translational isoprenyl addition) at its CaaX motif and subsequent loss of farnesyl through endoproteolytic cleavages, whereas lamin C lacks a farnesylation site altogether [8]. The zinc metalloproteinase STE24 (ZMPSTE24), which is highly expressed in the heart, is responsible for lamin A maturation via proteolytic cleavage [9]. In contrast, farnesylation is maintained in B-type lamins [10]. The expression of A-type lamins is influenced by matrix stiffness [11], which directs cell differentiation towards either a stiff and contractile lineage or soft lineage, depending on A-type lamins content [12]. In contrast, B-type lamin expression patterns are not regulated in a stiffness-dependent manner [11]. See Fig. 1 for a graphical representation of the structure and expression pattern of lamins.

Mechanosensitive pathways in cardiolaminopathy constitute a complex and challenging medical puzzle. *LMNA*-related heart failure (HF) presents a broad spectrum of clinical manifestations, ranging from conduction problems, atrial and ventricular arrhythmias, and atrioventricular block to isolated cardiac dysfunction, often leading to devastating outcomes [13]. While treatment advancements have significantly improved the prognosis for most forms of HF, individuals afflicted by inherited forms of DCM, prominently featuring *LMNA* mutations, continue to face grim prospects. The continuous interaction between cells and their extracellular matrix (ECM) intricately shapes both the biomechanical and biochemical characteristics of the ECM. Simultaneously, this interaction profoundly impacts cellular functionality by activating signal transduction pathways responsible for governing gene and protein expression [14]. Here, we summarise the enigma of cardiolaminopathy, mainly focusing on the mechanosensitive pathways and their downstream effectors. Furthermore, we aim to shed light on the genetic and molecular complexity of cardiolaminopathy, highlighting the urgent necessity to uncover novel therapeutic targets. Unraveling and redefining the potential modifications in the pathways are essential mechanisms in the gene regulation of mechanotransduction. There is an increasing demand to speed up research from fundamental to preclinical studies to address the needs of individuals affected by this devastating condition.

To improve the accuracy and reliability of future research, it is essential that we take into account the inconsistency with which protein and pathway names were represented in previous studies. Our review aims to address discrepancies in existing research by providing a thorough alignment of protein and pathway names with correct gene nomenclature.

### Abnormal mechanotransduction in cardiolaminopathy

Given the location of the nuclear lamina, it is hardly surprising that A-type lamins connect the nuclear interior to the cytoskeleton. A-type lamins are part of a linker between the cytoskeleton and nucleoplasm complex (LINC), which spans the nuclear envelope (NE) [15]. The key elements in this complex are SUN domain proteins at the inner nuclear membrane that extend into the perinuclear space, where they link to and localize nesprins (SYNE1 and SYNE2) at the outer nuclear membrane [15]. On the cytoplasmic side, nesprins link to cytoskeletal elements as they can bind actin directly [16] and indirectly connect to microtubules and IFs [17]. On the nucleoplasmic side, the SUN domain protein SUN1 has a high affinity for lamin A [18]. Lamins can bind chromatin directly. A-type lamins are involved in many biological processes, such as nuclear patterning, and regulation of large chromatin domains called lamina-associated domains (LADs) [19]. LADs are localized at gene-poor heterochromatic regions, and genes localized at LADs are often repressed [20]. A-type lamins also interact with chromatin-binding proteins, such as the LAP2-emerin-MAN1-containing (LEM) domain in emerin (EMD). The LEM domain binds to the chromatin protein BAF (BANF1), which undergoes a conformational change during self-assembly of the emerin N-terminal region [21–23]. On the cytoplasmic side, nesprins link to cytoskeletal elements as they can bind actin directly [16] and indirectly connect to microtubules and IFs [17]. In the myocardium and skeletal muscle, nesprins also connect to the Z-discs of the contractile sarcomeres [16, 21].

More than a decade ago, it was determined that the LINC complex is not only a physical connection but also a mechanosensor, as it was discovered that the sun-nesprin interaction is critical for cellular mechanotransduction and cellular tension [24]. Thus, the LINC complex is part of the mechanotransduction pathway and transduces mechanical forces to the nucleus. Signals from the environment are transmitted to ECM molecules that enter the cell through integrins and are transmitted to actin fibers via focal adhesion molecules and further into the nucleus via LINC molecules [25]. The perinuclear actin cap, which forms around the nucleus in a LINC-mediated process, is much more sensitive to mechanical stresses than stress fibers. Therefore, rapid mechanosensitive transduction is required between the environment and the nucleus to protect and properly exert a mechanoresponse [26].

The increased expression of stiff matrices suggests that A-type lamins have an important role in the mechanotransduction of extracellular mechanical signals into appropriate cellular responses [27]. Other roles for lamins include maintenance of NE stiffness and integrity [28]. B-type lamins are associated with nuclear elasticity in this role, while A-type lamins are responsible for viscous characteristics [11, 29]. Lamins also regulate gene expression through binding (hetero)chromatin at lamin-associated domains and interacting with various transcription factors [5]. Indeed, many of lamin's functions are performed indirectly through interactions with proteins [30, 31]. Therefore, abnormal lamins could lead to severe interruptions to biological and mechanosensitive processes.

Given that the nucleus functions as a mechanosensor through its connection to the cytoskeleton and, consequently, to the ECM via the LINC complex [30], cardiolaminopathy results in substantial disruption of nuclear mechanobiological processes. The degree of these disruptions is directly associated with the overall clinical severity of the phenotype [32]. Abnormal lamina contributes to an abnormal LINC complex, an imbalance between extracellular and intracellular tension, and, as a consequence, impaired mechanotransduction [33]. Uncoupling of the nucleus from the cytoplasm results in the inability to properly sense and respond to matrix stiffness: it was shown that regardless of matrix stiffness, *LMNA* pathogenic variant-carrying striated muscle cells behaved as if they were on a rigid matrix by expressing more stress fibers, vinculin (*VCL*), and focal adhesion kinases than wild-type cells [34]. In three *Lmna* mouse models and muscle biopsies from individuals with *LMNA*-related muscular dystrophy, the mutations destabilized the nucleus, causing temporary nuclear envelope ruptures in muscle cells. This led to DNA damage, activation of DNA damage responses, and reduced cell viability [35]. Furthermore, it was demonstrated that mutant cells fail to adapt to mechanical stress, as evidenced by unchanged focal adhesions and damage to the actin filaments in the cytoskeleton. The protein reflecting this abnormal response compared to wild-type (WT) myoblasts is Yes-associated protein (YAP/YAP1) [34].

### Hippo pathway

#### The regulation and function of YAP1

**Fig. 2:**
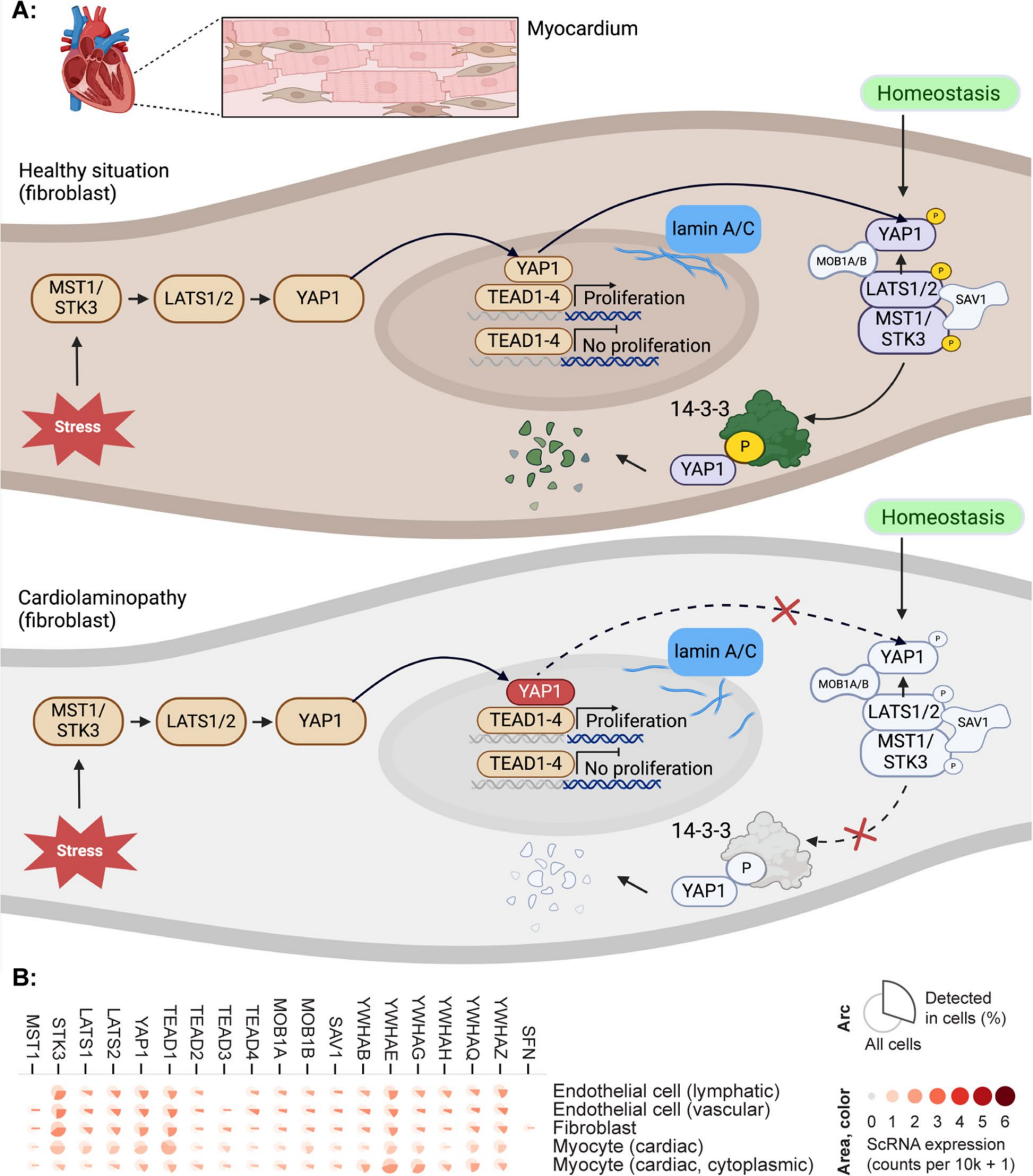
A schematic overview of the Hippo signaling pathway. **A** The laminopathic cell experiences stress, and activate the canonical Hippo pathway, resulting in the YAP1-TEAD complex to drive the transcription of proliferative genes. During homeostasis, residue-phosphorylation generates a 14–3-3-protein binding site, causing cytoplasmic sequestration and marking them for proteasomal degradation, ultimately inhibiting YAP and WWTR1 (previously known as TAZ) activity and partial degradation of YAP1. Note that the 14–3-3 protein family is encoded by six genes (YWHAB, YWHAE, YWHAG, YWHAH, YWHAQ, YWHAZ, and SFN) **B** Single-cell RNA expression profiles of genes encoding crucial proteins of the Hippo pathway in selected myocardial cell types (source: Multi-Gene Single Cell Viewer from GTEx). While the displayed genes are considered to be involved in the Hippo pathway and laminopathy, only some of them show sufficient RNA expression in cardiac cells (e.g., see the difference between paralogues MST1 and STK3) and might have different involvement in cardiolaminopathy

WW domain containing transcription regulator 1 (WWTR1), previously also known as transcriptional coactivator with PDZ-binding motif (TAZ, note: it should not be confused with phospholipid-lysophospholipid transacylase TAFFAZIN, previously also known as TAZ), binds to YAP1. Together, they are the effectors of an important pathway implicated in mechanotransduction, namely the Hippo pathway [36]. YAP1 and WWTR1 (YAP1/WWTR1) are considered both sensors and mediators of mechanical cues originating from the cell environment [37]. First identified in Drosophila, the core (also referred to as the cassette) of the Hippo pathway, composed of Hippo (Hpo), Salvador (Sav), Warts (Wts), and Mob-as-tumour-suppressor proteins, as well as the effector Yorkie (Yki) [38], is highly conserved in mammals [39]. The mammalian pathway consists of an enzymatic cascade of two main kinase families, Hpo orthologs macrophage stimulating 1 (MST1) and serine/threonine kinase 3 (STK3, previously known as MST2) and Wts orthologs large tumor suppressor kinase 1/2 (LATS1/2) [40], the activation of which results in the inactivation of YAP1/WWTR1 [41]. MST1/STK3 can be activated by autophosphorylation or via phosphorylation by TAO kinases (TAOK1/2/3) [42]. When active, MST1/STK3, along with the adaptor protein salvador family WW domain-containing protein 1 (SAV1, Sav ortholog), phosphorylates MOB kinase activators MOB1A/1B (Mats orthologs) [36], which bind to LATS1/2, phosphorylating the latter [43]. Phosphorylated LATS1/2 can phosphorylate YAP1/WWTR1 (Yki ortholog) [41]. The phosphorylation of YAP1 by LATS1/2 occurs at Ser127 residue, and phosphorylated YAP1 is subsequently inactivated and sequestered in the cytoplasm through the binding to 14–3-3 proteins and, thereby, inhibiting its function by conducting the complex towards proteasomal degradation [42]. In contrast, if the Hippo cassette is inactive, YAP1/WWTR1 remains unphosphorylated and is able to translocate into the nucleus. YAP1/WWTR1 lacks deoxyribonucleic acid (DNA) sequence-specificity. Therefore, to stimulate the transcription of target genes, they must bind to sequence-specific transcription factors, the most common of which is the TEAD group (containing genes *TEAD1/2/3/4*, Scalloped ortholog) [36]. See Fig. 2 for more details on the function and expression of the Hippo pathway genes.

Targets of YAP1 are involved in mitogenic activity necessary for cell proliferation, survival, and tissue growth [44]. In adult myocardium, overexpression of unphosphorylated YAP1 leads to cardiomyocyte (CM) proliferation, thickening of ventricular walls, and improvement in cardiac function, effectively inducing a fetal cell state in adult cells [45], corresponding to the postnatal expansion of the cardiac regeneration window [46]. Additionally, the Hippo pathway is a convergence point of cellular signaling interacting with multiple major pathways, including Wnt/β-catenin, insulin-like growth factor, phosphoinositide 3-kinase mediated phosphorylation of AKT1 (RACα serine/threonine-protein kinase or PI3K-RACα/Akt), and mTOR (MTOR) signaling [47].

#### Mechanosensing abilities and non-canonical regulation of YAP1 activity

Next to regulation via the phosphorylation of the Hippo pathway, non-canonical regulation of YAP1 activity has been described [37]. YAP1/WWTR1 activation and translocation into the nucleus can be induced through mechanical forces coming from the cell environment [48], illustrating YAP1/WWTR1 mechanosensing abilities. ECM stiffness is an important factor in dictating YAP1/WWTR1 subcellular shuttling. On soft matrices, YAP1 activity is inhibited due to nuclear exclusion, while stiff matrices have enhanced YAP1 activity and cell spreading [37]. The stiffness-dependence of YAP1 has been demonstrated using engineered fluorescent reporter genes coupled with YAP1 target promoters [49]. This study confirmed that soft matrices favor cytoplasmic localization and inactivation of YAP1, while on stiff substrates, nuclear aggregation and fluorescent reporter expression indicative of YAP1 activation were observed. Notably, rather than the ECM itself, cytoskeletal elements are paramount in regulating YAP1 localization [50]. Using a nanopillar measurement method, it was revealed that YAP1 translocation into the nucleus is guided by force-induced displacements in the perinuclear region dominated by the actin cap [51].

The aforementioned YAP1-induced cell spreading coincides with Ras homolog family member A (RHOA/Rho) activation, which regulates stress fibers, actin bundles, and actomyosin structures [41, 51]. Inhibition experiments showed that RHOA activation and cytoskeletal tension, which can be characterized by stress fibers, are necessary for YAP1 nuclear activity. Further, RHOA stimulates the nuclear import of TAZ [36]. Therefore, it has been shown that non-canonical regulation of YAP1/WWTR1 is involved in the translation of mechanical cues by YAP1/WWTR1 rather than the Hippo pathway. Indeed, RHOA inhibition reinforces the “soft state” response from the cell, resulting in YAP1/WWTR1 inactivation [52]. Moreover, RHOA inhibition can activate LATS, revealing the involvement of mechanical cues in the activity of the canonical Hippo pathway [52]. The precise role of the Hippo pathway connected to mechanotransduction is being revealed slowly. The inhibitory role of the Ras-related GTPase RAP2 (RAP2A) in inhibiting YAP1/WWTR1 nuclear entry was studied, revealing that ECM stiffness communicates via focal adhesions to downstream RAP2A, which in turn activates LATS via intermediates [53].

#### YAP1 involvement in cardiac remodeling

The aberrant localization of YAP1 is a feature of *LMNA* mutants and is associated with faulty mechanotransduction. However, it remains unknown how the Hippo pathway regulates cardiac remodeling in different pathologies [34, 54]. Upregulation of YAP1 expression and its increased nuclear levels have been reported in failing human hearts [54]. YAP1 knockout mediates the development of DCM, while hypertrophic cardiomyopathy (HCM) involves a deficiency in Hippo signaling, the upregulation of YAP1 transcript and protein levels, as well as the downregulation of its Ser127 phosphorylation [46, 54]. This sequence of events leads to increased transcription levels of target genes, including myosin heavy chain 7 (*MYH7)* and troponin T2 cardiac type (*TNNT2)* [55]. YAP1 inactivation correlates with decreased proliferation of CMs and cardiac hypoplasia [44]. Interestingly, it has been suggested that LATS can regulate hypertrophic cues independently of YAP1, suggesting that YAP1 is more important for CM survival and regulation than hypertrophic growth [44]. Thus, the involvement of Hippo and YAP1 in cardiomyopathies is unclear and likely mutation- and tissue-specific, warranting further investigation. Some indirect clues have been gathered through the *Lmna* H222P/H222P murine model, as it has been shown that treatment with an angiotensin-converting enzyme (ACE) inhibitor improves fractional shortening [56], reaffirming that angiotensin II (Ang2) encoded by angiotensinogen (AGT) contributes to cardiac remodeling. Since YAP1 is known to regulate the transdifferentiation of fibroblasts into myofibroblasts in the presence of Ang2 [57], it could be involved in this type of remodeling. Thus, levels of Ang2 and YAP1 should be studied together in one model to investigate whether YAP1 contributes to DCM-related fibrosis through its effects on Ang2.

#### Impaired YAP1 nuclear translocation

Lamins impact the formation of the actin network, which induces force-dependent nuclear entry of YAP1. It has been shown that disruption of actin cap formation, known to occur in *LMNA* null cells, impairs YAP1 translocation [51]. Beyond actin, a damaged nuclear lamina itself can alter YAP1 import. The severity of structural alterations caused by defective lamina can be a determinant of YAP1 localization. In murine *Lmna* delK32/ + mutant cells, YAP1 exclusion from the nucleus did not occur at high cell density in mesenchymal stem cell (MSC) cultures despite activating the upstream Hippo pathway [58]. In contrast, an appropriate density-dependent YAP1 exclusion was noted in human *LMNA* H222P/H222P MSCs [58], which indicates the mutation-dependent influence of lamins on nuclear transport complexes. Moreover, besides YAP1 phosphorylation by the Hippo pathway, non-canonical factors like SRC proto-oncogene, non-receptor tyrosine kinase (SRC)-mediated phosphorylation at different sites are now recognized as essential for YAP1 activation [36]. Src kinases, whose activation is connected to the state of the cytoskeleton, mediate the nuclear localization of YAP1 and suppress export to the cytoplasm [59]. In mutation-carrying cells, Src-dependent phosphorylation continued at high cell density [58]. This further indicates that in certain mutations, non-canonical regulation favors inappropriate YAP1 activation.

#### Non-canonical MST1 involvement in DCM

Besides its regulatory role on YAP1/WWTR1, non-canonical functions of MST1 have been connected to mechanisms that are impaired in cardiomyopathies [55, 60, 61]. In the context of DCM-associated fibrosis, MST1 has been shown to phosphorylate the BCL2 apoptosis regulator/B-cell lymphoma extra-large protein (Bcl-xL/BCL2L1), an anti-apoptotic agent [60]. The phosphorylation of BCL2L1 at Ser14 correlated with increased myocardial apoptosis and fibrosis in DCM [60]. Aside from promoting apoptosis in DCM, MST1 is also involved in suppressing autophagy [61], the process of lysosomal recycling of cellular components. Notably, while DCM features an up-regulation of MST1 phosphorylation, phosphorylation is down-regulated in HCM [61], indicating that MST1-mediated autophagy inhibition is higher in DCM. Lending support to this, MST1 expression is down-regulated in HCM via PI3K/AKT1-mediated forkhead box O3 (FOXO3) inhibition [55], leading to increased YAP1 activation. In turn, YAP1 enhances AKT1, forming a positive feedback loop, which leads to increasingly lower MST1 expression and higher autophagy [55], promoting cell survival. As such, HCM is accompanied by increasingly lower MST1 levels, which could affect autophagy in the opposite manner from DCM.

Alongside YAP1 and lamins, myocardin-related transcription factor A (MRTFA), also known as megakaryoblastic leukemia 1 protein (MKL1), is often used to study mechanosensing and transduction [62, 63]. MRTFA is a mechanosensitive transcription factor whose localization from cytoplasm to nucleus is decreased in DCM [64]. MRTFA mediates the activation of serum response factor (SRF), which is involved in the differentiation of cardiac lineages, including CMs, and cardiac morphogenesis [65]. When CMs are subjected to mechanical stress, the serum response factor (SRF) activation triggers the hypertrophic program [66]. Here, actin polymerization and its control by RHOA influence the MRTFA localization [64]. This has been observed using mouse models, namely *Lmna*-/- and *Lmna* N195K/N195K, which have a disorganized actin network and decreased nuclear presence of MRTFA [67]. Therefore, mutant lamin-associated actin network impairments result in decreased nuclear translocation of MRTFA and subsequent lower activation levels of SRF, which impairs the growth and differentiation of CMs.

Further, in addition to abnormal YAP1 localization in myogenic precursors carrying an *LMNA* mutation, the localization of MRTFA was impaired [34]. In studies of MSC lines, the strain to which the cell is subjected affects the subcellular localization of MRTFA and the amount of protein expressed [68]. This contrasts with YAP1, which was only affected in terms of localization. As mechanotransduction is impaired in *LMNA* mutants, it is interesting to study whether the amount of MRTFA is subject to change in every *LMNA* mutant and to what extent.

### MAPK pathway

**Fig. 3:**
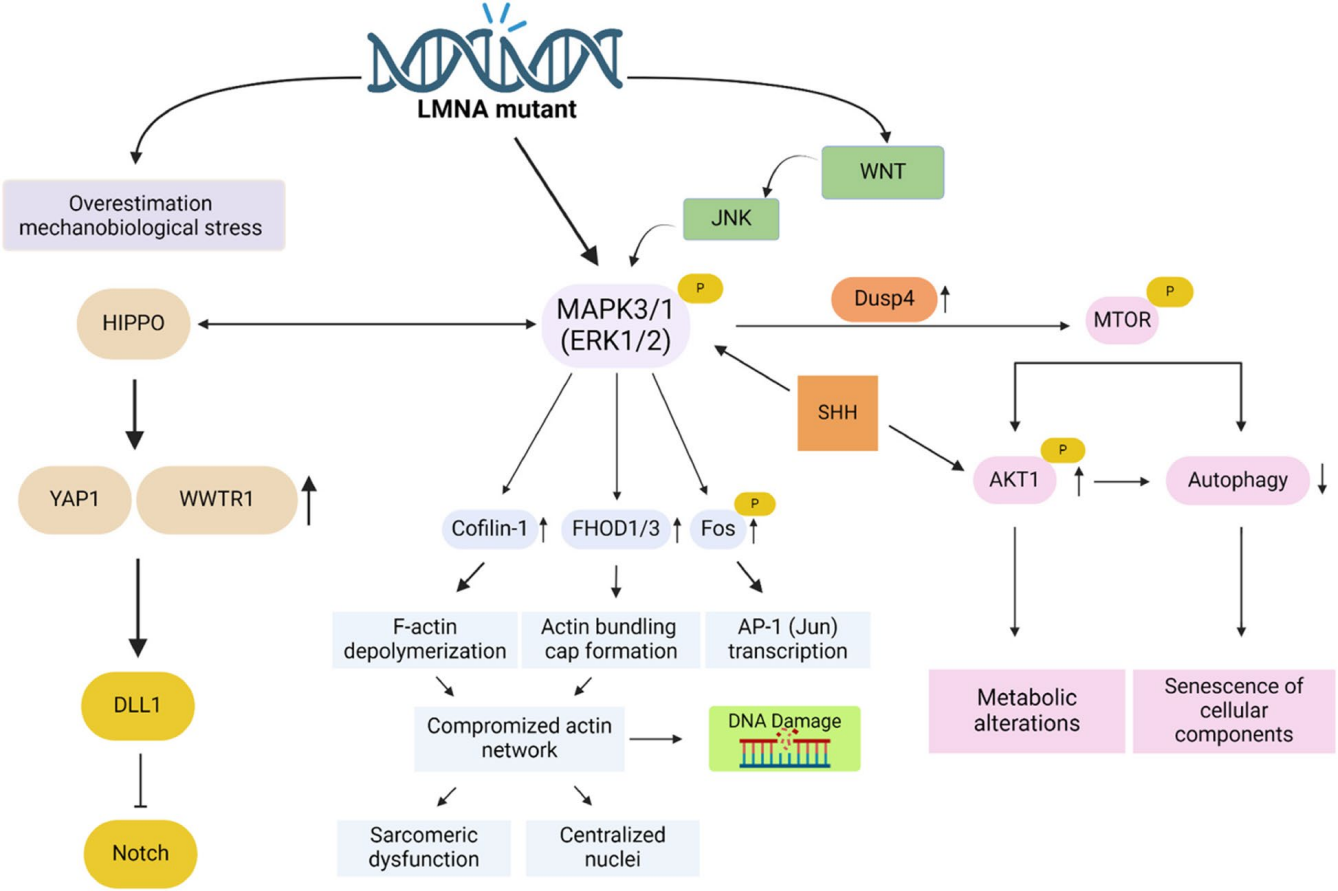
A schematic overview of LMNA mutant pathways and MAPK3/1 (ERK1/2) initiated changes in cardiac remodeling. Altered inter- and intracellular pathways lead to cellular phenotypic differences, giving rise to typical LMNA hallmarks, such as DNA damage, impaired actin cap and alterations in mechanoresponses

The role of mitogen-activated protein kinase (MAPK) pathways in cardiac remodeling has been studied extensively using rodent models for cardiolaminopathy. In the cardiac tissue from *Lmna* H222P/H222P mice, a common mode for cardiolaminopathy [69], hyperactivation of MAPK pathways occurs prior to the appearance of cardiomyopathy [70]. Activation and, even more importantly, the crosstalk of MAPK3/1 (also known as ERK1/2) [70], MAPK8 (also known as JNK) [70], and MAPK14 (also known as p38) [71] branches, as well as the expression of selected target genes, was increased, which in turn could alter the cytoskeletal architecture or several other cellular processes [72, 73]. This activation occurred prior to the detection of fibrotic markers, indicating that MAPK activation is causative in cardiolaminopathy-associated remodeling. After this first detection of hyperactivation of the MAPK3/1 (ERK1/2) branch of MAPK signaling in cardiolaminopathy [70], the road was paved for investigation concerning the involvement of this pathway in disease progression. The following section is dedicated to summarising the current knowledge of MAPK3/1 (ERK1/2) involvement in dilatation and fibrotic remodeling in DCM. A schematic overview of possible events contributing to characteristic events of cardiolaminopathy is provided in Fig. 3.

#### MAPK3/1 (ERK1/2) in the development and progression of remodeling

Typically, MAPK3/1 (ERK1/2) is associated with a hypertrophic response. The hyperphosphorylated p-MAPK3/1 (ERK1/2) in DCM could indicate the degree of activation that may influence the effects of this pathway [72] and determine whether progression towards DCM or HCM occurs. The role of the hyperactive MAPK3/1 (ERK1/2) pathway in cardiolaminopathy has been studied using pharmacological inhibition [74], which normalized the expression of the remodeling- and HF-associated natriuretic peptides A/B (NPPA/B). Further, MAPK3/1 (ERK1/2) inhibition resulted in the activation of genes involved in sarcomere organization and prevented left ventricular (LV) dilatation from developing [74]. A MEK1/2 inhibitor, acting upstream of ERK, has similar effects in preventing end-stage fibrosis [75]. This approach lent evidence to the claim that the hyperactivation of MAPK3/1 (ERK1/2) signaling is involved in the development of ventricular remodeling. In induced pluripotent stem cells-derived cardiomyocytes (iPSC-CMs), it was noted that MAPK3/1 (ERK1/2) blockade attenuates apoptosis due to electrical stimulation [76]. Additionally, MAPK3/1 (ERK1/2) seems to be active in disease progression, as its pharmacological inhibition following the appearance of disease markers resulted in an improvement in cardiac measures [77].

In addition, the effects of MAPK3/1 (ERK1/2) hyperactivation in EDMD-related DCM in a mouse model have been confirmed using a germline deletion of MAPK3/ERK1 [72]. It was shown that the preventative influence on DCM development was abolished by 20 weeks of age, with an upregulation of MAPK1/Erk2 compensating for the depletion of MAPK3/Erk1 [72]. It is important to keep this in mind when developing Erk-inhibiting pharmaceuticals to avoid the waning of effects over time.

To explain the increase in MAPK3/1 (ERK1/2) target expression, it has been suggested that the nuclear lamina acts as a scaffold for MAPK3/1 (ERK1/2). It was shown that A-type lamins act as a scaffold for MAPK3/1 (ERK1/2) and the fos proto-oncogene, AP-1 transcription factor subunit (FOS, also known as c-FOS), which is part of the jun proto-oncogene, AP-1 transcription factor subunit (JUN/ also known as AP1) [78]. Active MAPK3/1 (ERK1/2) interacts with A-type lamins in the nuclear lamina, where it induces FOS phosphorylation, facilitating FOS’ release from the NE and enhancing JUN-mediated transcription [78]. Until recently, it was unknown how an *LMNA* variant could affect this interaction. In the *Lmna* H222P/H222P mouse model, mutant lamins sequestered MAPK3/1 (ERK1/2) regardless of phosphorylation status, resulting in a proportional increase of active p-MAPK3/1 (ERK1/2) at the nuclear periphery [79]. This could increase interaction with molecules such as FOS and enhance the transcriptional activity of MAPK/Erk targets.

Besides the myocardium, MAPK3/1 (ERK1/2) hyperactivation in EDMD mouse models showed pathological effects in skeletal muscle [80]. Thus, given that striated tissue is affected, it seems that physiological stress, the common denominator found in cardiac and skeletal muscle, is required for MAPK3/1 (ERK1/2) activation. As the Hippo pathway is a convergence point for many other signaling pathways, it is likely that abnormal activities in these affect YAP1 activity. While a connection between YAP1 and MAPK3/1 (ERK1/2) has not been investigated in cardiolaminopathy, findings from non-small cell lung cancer cells indicate a positive correlation between the level of MAPK3/1 (ERK1/2) activation and YAP1 protein and target gene, including the cellular communication network factor 2 (CCN2, also known as CTGF) transcription levels [81]. Additionally, YAP1 overexpression improves septic cardiomyopathy by mediating increased MAPK3/1 (ERK1/2) activity [82]. As such, YAP1 involvement in MAPK3/1 (ERK1/2) activity and vice versa in *LMNA*-related cardiac remodeling is a promising research avenue.

In line with the notion that YAP1 and MAPK/ERK activities are related, affected mechanotransduction results in an overestimation of mechanical stress [34], leading to a translocation of YAP1, which in turn increases MAPK3/1 (ERK1/2) signaling. The hyperactivation of MAPK3/1 (ERK1/2) could further impair cytoskeletal networks. Investigation of several *LMNA* mutations that feature MAPK3/1 (ERK1/2) hyperactivation revealed that p-MAPK3/1 (ERK1/2) interacts with and catalyzes the activation (by phosphorylation) of cofilin 1 (CFL1), which then participates in the depolymerization of F-actin filaments in CM sarcomeres, resulting in LV dysfunction [83].

Another link between MAPK3/1 (ERK1/2) and impaired mechanotransduction is inaccurate nuclear positioning. LINC complexes assemble into transmembrane actin-associated nuclear (TAN) lines, which connect the nucleus to actin filaments via lamins, allowing for rearward nuclear movement [84]. Proteins with formin domains are involved in forming actin bundles, a critical process for establishing routes for intracellular migration [85]. The internalization or centralization of nuclei has been observed in *Lmna* H222P/H222P mice [80, 86] and recently connected to the hyperactivation of MAPK3/1 (ERK1/2) [87]. MAPK3/1 (ERK1/2) hyperactivation phosphorylates formin homology 2 domain containing 1/3 (FHOD1/3) protein, negatively affecting FHOD1/3 ability to enhance actin bundling in striated muscle, which could impair the stress-resistance of actin fibers required for nuclear displacements. The early involvement of nuclear positioning in sarcomere formation could also play a role in cardiomyopathy [87]. Apart from the role of MAPK3/1 (ERK1/2) in nuclear positioning, the role of mutant lamins in TAN line function requires further study since it has been shown that lamin depletion abrogates the anchoring of TAN and, therefore, force transmission [84].

#### MAPK3/1 (ERK1/2) and MAPK14 (p38) involvement in autophagy through AKT1/MTOR

In both *LMNA* H222P mouse models and patients, the activation of the AKT1/MTOR pathway, as well as the phosphorylation of MAPK3/1 (ERK1/2), is increased prior to the manifestation of clinical features of DCM [88]. AKT1 and MAPK3/1 (ERK1/2) converge on MTOR, resulting in activation and subsequent inhibition of autophagy [88]. Further, dual specificity phosphatase 4 (DUSP4) is upregulated by MAPK3/1 (ERK1/2) and was shown to result in DCM via metabolic disturbances and autophagy inhibition through the AKT1/MTOR pathway [89], pointing to the complex interplay of MAPK3/1 (ERK1/2) and AKT1 in autophagy. In a follow-up study, DUSP4 upregulation enhanced the expression of HF and fibrosis markers and LV dysfunction [89]. Notably, in WT cells, DUSP4 activity is self-limiting due to its inhibitory action on MAPK3/1 (ERK1/2). However, mutant lamins may disturb interactions between these molecules, leading to a positive feedback loop [79, 89].

It is evident that MAPK3/1 (ERK1/2) hyperactivation has several mechanistic contributions to aberrant cellular processes relevant to the pathogenesis of DCM. However, some important shortcomings and limitations make it difficult to discern the exact mechanism of MAPK3/1 (ERK1/2) in DCM. Firstly, the bulk of knowledge is based on a murine *Lmna* H222P/H222P model [86]. Although the model recapitulates DCM, differences between humans and mice might limit translatability. Additionally, for comparison to DCM in humans, tissue sections have often been used [75]. These are usually harvested from post-transplantation HF patients, representing only the end-stage of the disease and offering the limited possibility to be used as proper controls. Furthermore, fibrosis and LV dilatation are often investigated in conjunction, as is apparent from the previous discussion. This is a natural approach, given the interdependent relationship between cardiomyocytes and cardiac fibroblasts, which results in the co-occurrence of these phenotypes.

The hyperactivation of MAPK14/p38 was also found in *LMNA* mutation carriers, and the inhibition of this signaling branch prevented LV dilatation [71, 90]. Thus, a widespread activation of MAPK pathways is seen in DCM development. While the exact connection to lamins is still unknown, it is thought that the mechanical stress encountered by contractile cells could activate MAPK branches inappropriately in *LMNA* mutants [71, 90].

#### MAPK8 (JNK) pathway inhibition in heart failure

JNK is known to be activated by various stress factors, including osmotic stress [91], which could be exacerbated by the diminished structural integrity of nuclei in lamin mutants. The *Lmna* H222P/H222P homozygous mutant mice demonstrating signs of DCM, show upregulation of the JNK pathway alongside MAPK3/1 (ERK1/2) [70]. Inhibition using the JNK pathway inhibitor SP600125 led to a reduction in nuclear length and an improvement in contractile dysfunction [92], and it can be inferred that MAPK8/JNK is implicated in both dilatation and fibrosis and the progression to HF. Like MAPK3/1 (ERK1/2), it was shown that pharmacological inhibition of MAPK8/JNK improved heart function after manifestation of the disease using the same murine model [77]. This indicates that JNK is involved in both the initiation and subsequent progression of DCM. Lamins might cause altered responses to stress, leading to the activation of the MAPK8/JNK pathway and the development of fibrosis through MAPK8/JNK interaction with JUN (AP1)-mediated collagen-1 regulation [92].

### Wnt/β-Catenin pathway

The Wnt/β-catenin pathway is well-known for its major role in guiding stem cells into specialized lineages. In the context of the cardiac mesoderm lineage, this process heavily relies on the precise regulation of Wnt signaling [93]. The Wnt family is encoded by 19 Wnt family member genes (WNT1-WNT16), and β-catenin is encoded by the catenin beta 1 (*CTNNB1*) gene. In mesenchymal stem cell differentiation, it was found that lamins have a role in mediating Wnt/β-catenin signaling through a direct interaction with β-catenin within the nucleus [94]. Further, lamin overexpression increased the nuclear entry of β-catenin, while decreased lamin expression had the opposite effect [94]. Thus, the appearance of lamins during differentiation could further affect differentiation direction by mediating Wnt/β-catenin signaling.

Postnatally, deficiency of Wnt/β-catenin signaling and upregulation of Frizzled proteins, which inhibit Wnt signaling, was found in *Lmna* H222P/H222P mice in correlation with ventricular remodeling and dysfunction, accompanied by conduction defects [95]. Improvement of diagnostic parameters was observed after activating Wnt signaling.

YAP1 can regulate Wnt signaling by directly interacting with β-catenin, which stimulates β-catenin/transcription factor 7 (TCF) transcriptional activity [44]. Further stimulation of Wnt signaling might be achieved through YAP1-mediated stimulation of insulin like growth factor 1 (IGF) signaling [44]. Wnt abnormalities are also found in other cardiac diseases, and it is possible that rather than being a cause, they are a consequence of underlying molecular events. Given the interactions between lamins, β-catenin, and YAP1, all of which can be affected in DCM, the contribution of Wnt/β-catenin remains a relevant area of study [95].

### Notch pathway

The Notch pathway, a well-known mechanotransduction cascade, is a key regulator of different cell fate functions such as proliferation, apoptosis, boundary formation, and regeneration [96]. The Notch pathway is indispensably involved in the cardiovascular system and has a pivotal role in the development and preserving homeostasis of the atrioventricular canal and valves, coronary vessels, and for growth and differentiation of the endocardium, myocardium, and epicardium [97]. Notch plays a role in several cardiomyopathies, e.g., congenital heart disease in the form of valve disease and DCM [98]. The Notch receptor 1 (NOTCH1) and its ligand jagged canonical Notch ligand 1 (JAG1) are especially found to be critical components of the development of cardiac stem cells (CSCs) into cardiac myocytes. NOTCH1 is involved in idiopathic DCM in children, and defects of NOTCH in CSCs cause severe attenuation in the generation of CMs [99]. It is known that mutations in presenilin 1/2 (*PSEN1/2*), which form part of the multi-subunit protease γ-secretase, which cleaves and releases the Notch intracellular domain, are involved in DCM with high pathogenicity [100]. In cardiac fibrosis, Notch acts as an inhibitor of the differentiation process of myofibroblasts. During fibroblast–myofibroblast transition, Notch receptors 1, 3, and 4 (NOTCH1/3/4) are down-regulated, indicating that Notch inhibition promotes myofibroblast formation [97]. It was shown that NOTCH1 has a controlling role in inhibiting myofibroblast proliferation and promoting the mobilization and expansion of CM precursor cells in mouse models [101]. Also, signaling crosstalk between YAP1/WWTR1 and Notch during myogenesis results in induced YAP1 translocation to the nucleus and mediated an upstream expression of the jagged canonical Notch ligand 2 (JAG2), followed by Notch activation during contractions [102, 103]. This indicates that during mechanical stress in skeletal muscle tissue, continuous stress is present, affecting the homeostasis of the YAP1-Notch equilibrium. In A-type lamin mutations of skeletal muscle, it was found that YAP1 was transcriptionally active despite activation of the Hippo pathway, which inhibits YAP1. Data showed a significant correlation between YAP1 deregulation, nuclear envelope defects, and disease severity [58]. The delta like canonical Notch ligand 1 (DLL1) was present in the serum of chronic HF failure patients, indicating that the Notch pathway affects diastolic function [104]. Increased levels of non-canonical Notch ligands DLL1 and periostin (POSN) were present in patients with DCM. Also, there is a strong correlation between diastolic dysfunction and the circulating levels of both proteins [105]. A link between Notch and progerin was found in Hutchinson-Gilford Progeria Syndrome (HGPS) cells expressing progerin. Progerin appears to activate several down regulators of the Notch pathway, such as Hes family BHLH transcription factor 1/5 (HES1/5) and Hes-related family BHLH transcription factor with YRPW motif 1 (HEY1) [106]. An altered Notch expression was found in mice carrying the R9C mutation in the *PLN* gene, which is a known initiator of DCM. In these mice, NOTCH1 and the ligand DLL1 were down-regulated, and delta-like canonical Notch ligand 1 (DLL3) was upregulated together with NUMB endocytic adaptor protein (NUMB), which resulted in proteasomal degradation of NOTCH1. NOTCH1 activators ADAM metallopeptidase domain 9/17 (ADAM9/17) were also upregulated, yet these high expression rates negatively inhibited Notch signaling [107]. These results conclude that NOTCH1 signaling in early-stage DCM is downregulated. Deficiencies in the Notch signaling inhibitor TIMP metallopeptidase inhibitor 3 (TIMP3) were found to cause DCM [107], which reveals novel avenues to explore.

### Hedgehog pathway

The Hedgehog pathway is a well-known regulator for cell differentiation, tissue polarity, and cell proliferation and is critical for maintaining tissue polarity and stem cell population [108]. In terms of HF, Hedgehog enhanced neovascularization and prevented fibrosis after acute myocardial ischemia. Mainly, the sonic hedgehog signaling molecule (SHH) preserves cardiac muscle cells after myocardial infarction (MI) in a peroxide-induced oxidative stress environment [109]. These effects are partly responsible for the reduced extent of LV scarring and enhanced preservation of LV function in the chronic phase after MI. It was found that hedgehog receptor patched 1 (PTCH1) is expressed in cardiomyocytes, which is believed to be the instigator of the positive protective effects of hedgehog signaling [110]. MI reactivates the Hedgehog signaling pathway in adult mammals, upregulating the expression of angiogenic and cardioprotective genes, including vascular endothelial growth factor (VEGFA) [111]. VEGFA showed a relation between the Hedgehog stimulating of Notch via target gene expression under the influence of VEGFA [112]. Genetic therapy was tested with SHH in combination with a small molecule inhibitor AMD3100-stimulated progenitor-cell mobilization and showed promising results of reduced cardiac fibrosis and promoted the development of capillaries and smooth-muscle–containing vessels after MI [111]. Alterations in the Hedgehog pathways have been tested in several animal models. In a pig model, it was shown that SHH had a cardioprotective function by inducing nitric oxide (NO) production in fibroblasts and cardiomyocytes [113]. Further, SHH inhibition of NO was also observed via the SHH/PI3-K/AKT1 pathway in cultured rat endothelial cells and isolated primary coronary arteries [114]. As previously mentioned, coagulation factor II thrombin receptor (F2R, also known as PAR1) signaling seems to contribute to the development of Ang2-induced fibrosis. Therefore, it is interesting to realize that Hedgehog signaling controls Ang2 expression in both the embryonic heart and the adult heart (in the adult heart, Hedgehog signaling also controls Ang1). In relation to VEGFA, overexpression of Ang2 enhances coronary vessel growth [115]. BaLB/c mice were sensitive to Ang2-induced pressure overload, which exacerbated LV remodeling, damage, and, ultimately, DCM [116].

### Additional factors involved in mechanotransduction

#### TGFB1/2 (TGF-β) involvement

Nuclear accumulation of phosphorylated forms of SMAD family member 2 (SMAD2/3) proteins, effectors of transforming growth factor beta 1 (TGFB1, also known as TGF-β1), were elevated in *Lmna* H222P/H222P mice, suggesting to contribute to the fibrotic phenotype observed cardiolaminopathy [86]. Increased AKT1/MTOR phosphorylation has also been observed in relation to elevated transforming growth factor beta 2 (TGFB2) levels in patients [117]. Myoblast differentiation was found to be impaired, and collagen 1 production was upregulated, indicating activation of a fibrogenic program. The fibrogenic activity of TGFB1/2/SMAD2/3 was also described in an *Lmna*-silencing mouse model featuring both cardiac dilatation and fibrosis [118]. As mouse models of DCM exhibit prominent myocardial fibrosis, the TGFB2 connection to AKT1/MTOR seems to contribute to the DCM phenotype. TGF-β contribution to myocardial fibrosis in *Lmna* D300N mice has been linked to the activation of the DNA damage repair pathway and tumor protein 53 (TP53) [119]. This results in dysregulation of pathways involved in the cell cycle and apoptosis. TGF-β and cytokines were among the upregulated targets, which were reflected in myocardial fibrosis and increased non-myocyte population [119]. It was postulated that DNA damage repair and TP53 pathways are activated due to an unstable interaction between mutant lamins and the retinoblastoma protein, leading to the eventual activation of DNA damage repair and TP53 pathways [119]. Thus, impaired lamina interactions result in TGF-β upregulation and subsequent fibrosis. As noted, CCN2 (CTGF) is a fibrogenic growth factor. In *Lmna* H222P/H222P mice, higher activation of CCN2 (CTGF) was detected compared to wild-type mice, in conjunction with upregulation of TGFB1/2/SMAD2/3 signaling [120]. Mechanistically, TGFB1/2/SMAD2/3 likely upregulates MAPK3/1 (ERK1/2), which in turn mediated CCN2 (CTGF)activation prior to and after the detection of fibrosis in mutant hearts [120].

#### F2R/PAR and CCN2 (CTGF) in cardiac fibrosis

F2R (PAR1) signaling contributed to the development of Ang2-induced fibrosis, as Ang2 infusion led to cardiac fibrosis, inflammation, and remodeling [121]. This was attenuated in F2R (PAR1)-deficient mice where expression of pro-fibrotic markers was decreased, including reduced mRNA of CCN2 (CTGF) F2R (PAR1)-deficient mice compared to WT mice [121]. The precise role of F2R (PAR1) signaling in cardiolaminopathy still needs to be unraveled. Yet, it might be a more specific target than Ang2. ACE inhibitors are known to be less efficient than pathway-specific treatments. For example, a MAPK3/1 (ERK1/2) inhibitor, selumetinib, was more successful in improving DCM markers compared to an ACE inhibitor, benazepril, which is used as a standard cardiological treatment [56]. It is tempting to investigate the downstream targets of Ang2 signaling to determine novel approaches for *LMNA*-precision treatment.

Mice with silenced *Lmna* expression developed DCM, accompanied by interstitial fibrosis and inflammation. The expression of YY1 transcription factor (YY1, previously also known as Yin Yang 1) modulates the activity of bone morphogenetic protein 7 (BMP7) and CCN2 (CTGF) (up- and down-regulated, respectively), which act together to suppress fibrosis [118]. The proposed mechanism for fibrosis suppression could operate by BMP7 and CCN2 (CTGF) modulation-mediated TGFB1/2/SMAD2/3 signaling inhibition [118].

### Redox imbalances: reductive and oxidative stress as a possible second hit

*Lmna* H222P/H222P mice were found to have increased oxidative stress indicated by increased protein carbonylation, increased presence of cytochrome B-245 beta chain (CYBB, also known as NOX2), and decrease in the oxidative stress-countering enzyme glutathione synthetase (GSS) [122]. Additionally, *Lmna* H222P/H222P mice treated with a GSS precursor showed ameliorated cardiac function associated with decreased fibrosis. The mechanism for this is unknown, but it is tempting to speculate that *LMNA* mutations could lead to ventricular dilatation via the reactive oxygen species (ROS) system [122]. Recently, reductive stress has been studied in the context of myopathic *LMNA* mutations in a Drosophila model [123]. Mutant lamins in cells suffering from reductive stress aggregate in the cytoplasm, causing increased levels of sequestosome 1 (SQSTM1/P62), a coactivator of the NFE2 like BZIP transcription factor 2 (NFE2L2)/Kelch like ECH associated protein 1 (KEAP1) pathway, which regulates the expression of detoxification genes [123]. A later study that also used Drosophila models expanded on this topic by describing the nuclear enrichment of NFE2L2 in lamin mutant cells post cytoplasmic lamin aggregation and SQSTM1/P62 upregulation [124]. It was observed in the Drosophila melanogaster heart with the mutant lamin C gene expressed, that SQSTM1/P62 activated MTOR and inactivated protein kinase AMP-activated catalytic subunit alpha 2 (AMPK), leading to the activation of the PI3K/Akt/MTOR pathway, resulting in the inhibition of autophagy [124]. Thus, reductive stress, in combination with autophagy inhibition, could explain the development of cardiac dysfunction.

## Conclusions

Novel in vitro and in vivo models elucidated the importance of mechanobiological pathways in cardiolaminopathy. In the realm of mechanotransduction impairment, signaling pathways are pivotal in understanding force transduction at a molecular level. The interplay of the assessed pathways elucidates novel targets on the kinome level but also serves as validation for extensively studied cellular processes such as apoptosis and proliferation. Currently, inhibition of downstream effectors of mechanosensitive pathways has successfully been tested, both in vitro and in preclinical models. To date, it is feasible to inhibit and activate pathways in vivo, and some of these modulators were tested in clinical trials as potential future drugs to relieve symptoms of patients with cardiolaminopathy. The overview provided in this review paper gives a comprehensive summary of the molecular mechanisms underlying cardiolaminopathy.

### Supplementary Information

**Supplementary Material 1.**

## Data Availability

Information about gene expression was obtained from the Genotype-Tissue Expression (GTEx) portal: https://gtexportal.org/.

## Abbreviations

14–3-3: Group of regulatory proteins encoded by the YWHAB (ENSG00000166913), YWHAE (ENSG00000108953), YWHAG (ENSG00000170027), YWHAH (ENSG00000128245), YWHAQ (ENSG00000134308), YWHAZ (ENSG00000164924), and SFN (ENSG00000175793) gene
ACE: Angiotensin I converting enzyme
ADAM9: ADAM metallopeptidase domain 9 (ENSG00000168615)
ADAM17: ADAM metallopeptidase domain 17 (ENSG00000151694)
AGT: Angiotensinogen (ENSG00000135744, gene encoding Ang2)
AKT1: AKT serine/threonine kinase 1 (ENSG00000142208, also known as Act)
AMPK: Protein kinase AMP-activated catalytic subunit alpha 2 (ENSG00000162409)
Ang2: Angiotensin II
BANF1: BAF nuclear assembly factor 1 (ENSG00000175334, previous name: BAF)
BCL2L1: BCL2 like 1 (ENSG00000171552, previous name: BCL-XL)
BMP7: Bone morphogenetic protein 7 (ENSG00000101144)
CCN2: Cellular communication network factor 2 (ENSG00000118523, previous name: CTGF)
CFL1: Cofilin 1 (ENSG00000172757)
CMs: Cardiomyocytes
CSCs: Cardiac stem cells
CTNNB1: Catenin beta 1 (ENSG00000168036)
CYBB: Cytochrome B-245 beta chain (ENSG00000165168)
DCM: Dilated cardiomyopathy
DLL1: Delta like canonical Notch ligand 1 (ENSG00000198719)
DLL3: Delta like canonical Notch ligand 3 (ENSG00000090932)
DNA: Deoxyribonucleic acid
DUSP4: Dual specificity phosphatase 4 (ENSG00000120875)
ECM: Extracellular matrix
EDMD: Emery-Dreifuss muscular dystrophy
EMD: Emerin (ENSG00000102119)
F2R: Coagulation factor II thrombin receptor (ENSG00000181104)
F-actin: Polymeric form of actin encoded by the ACTC1, ACTA1, ACTA2, ACTG1, ACTG2 and ACTB genes
FHOD1: Formin homology 2 domain containing 1 (ENSG00000135723)
FHOD3: Formin homology 2 domain containing 3 (ENSG00000134775)
FOS: Fos proto-oncogene, AP-1 transcription factor subunit (ENSG00000170345)
FOXO3: Forkhead box O3 (ENSG00000118689)
GSS: Glutathione synthetase (ENSG00000100983, previous gene name: GSH)
HCM: Hypertrophic cardiomyopathy
HES1: Hes family BHLH transcription factor 1 (ENSG00000114315)
HES5: Hes family BHLH transcription factor 5 (ENSG00000197921)
HEY1: Hes Related Family BHLH Transcription Factor With YRPW Motif 1 (ENSG00000164683)
HF: Heart failure
HGPS: Hutchinson-Gilford progeria syndrome
IF: Intermediate filament
IGF: Insulin like growth factor 1 (ENSG00000017427)
iPSC-CMs: Induced pluripotent stem cells-derived cardiomyocytes
JAG1: Jagged canonical Notch ligand 1 (ENSG00000101384)
JAG2: Jagged canonical Notch ligand 2 (ENSG00000184916)
JUN: Jun proto-oncogene, AP-1 transcription factor subunit (ENSG00000177606, previous gene name: AP1)
KEAP1: Kelch like ECH associated protein 1 (ENSG00000079999)
LAD: Lamina-associated domain
LATS1: Large tumor suppressor kinase 1 (ENSG00000131023)
LATS2: Large tumor suppressor kinase 2 (ENSG00000150457)
LEM: LAP2-emerin-MAN1-containing domain
LINC: Linker of cytoskeleton and nucleoplasm complex
LMNA: Lamin A/C (ENSG00000160789)
LMNB1: Lamin B1 (ENSG00000113368)
LMNB2: Lamin B2 (ENSG00000176619)
LV: Left ventricle
MAPK: Mitogen-activated protein kinase (large regulatory protein family)
MAPK1: Mitogen-activated protein kinase 1 (ENSG00000100030, previous name: ERK2)
MAPK3: Mitogen-activated protein kinase 3 (ENSG00000102882, previous name: ERK1)
MAPK8: Mitogen-activated protein kinase 8 (ENSG00000107643, previous name: JNK)
MAPK14: Mitogen-activated protein kinase 14 (ENSG00000112062, previous name: p38)
MI: Myocardial infarction
MRTFA: Myocardin related transcription factor A (ENSG00000196588, previous gene name: MKL1)
MOB1A: MOB kinase activator 1A (ENSG00000114978)
MOB1B: MOB kinase activator 1B (ENSG00000173542)
mRNA: Messenger ribonucleic acid
MSC: Mesenchymal stem cell
MST1: Macrophage stimulating 1 (ENSG00000173531)
MTOR: Mechanistic target of rapamycin kinase (ENSG00000198793, also known as: mTOR)
MYH7: Myosin heavy chain 7 (ENSG00000092054)
NE: Nuclear envelope
NFE2L2: NFE2 like BZIP transcription factor 2 (ENSG00000116044)
NO: Nitric oxide
NOTCH1: Notch receptor 1 (ENSG00000148400)
NOTCH3: Notch receptor 3 (ENSG00000074181)
NOTCH4: Notch receptor 4 (ENSG00000204301)
NUMB: NUMB endocytic adaptor protein (ENSG00000133961)
NPPA: Natriuretic peptide A (ENSG00000175206)
NPPB: Natriuretic peptide B (ENSG00000120937)
POSTN: Periostin (ENSG00000133110)
PSEN1: Presenilin 1 (ENSG00000080815)
PSEN2: Presenilin 2 (ENSG00000143801)
PTCH1: Patched 1 (ENSG00000185920)
RAP2A: RAP2A, member of RAS oncogene family (ENSG00000125249, previous gene name: RAP2)
RHOA: Ras homolog family member A (ENSG00000067560)
ROS: Reactive oxygen species
SAV1: Salvador family WW domain containing protein 1 (ENSG00000151748)
SHH: Sonic Hedgehog signaling molecule (ENSG00000164690)
SMAD2: SMAD family member 2 (ENSG00000175387)
SMAD3: SMAD family member 3 (ENSG00000166949)
SQSTM1: Sequestosome 1 (ENSG00000161011, previous gene name: P62)
SRC: SRC proto-oncogene, non-receptor tyrosine kinase (ENSG00000197122)
SRF: Serum response factor (ENSG00000112658)
STK3: Serine/threonine kinase 3 (ENSG00000104375, previous gene name: MST2)
SUN1: Sad1 and UNC84 domain containing 1 (ENSG00000164828)
SYNE1: Spectrin repeat containing nuclear envelope protein 1 (ENSG00000131018)
SYNE2: Spectrin repeat containing nuclear envelope protein 2 (ENSG00000054654)
TP53: Tumor protein P53 (ENSG00000141510)
TEAD1: TEA domain transcription factor 1 (ENSG00000187079)
TEAD2: TEA domain transcription factor 2 (ENSG00000074219)
TEAD3: TEA domain transcription factor 3 (ENSG00000007866)
TEAD4: TEA domain transcription factor 4 (ENSG00000197905)
TAOK1: TAO kinase 1 (ENSG00000160551)
TAOK2: TAO kinase 2 (ENSG00000149930)
TAOK3: TAO kinase 3 (ENSG00000135090)
TAN: Transmembrane actin-associated nuclear lines
TCF: Transcription factor 7
TGFB1: Transforming growth factor beta 1 (ENSG00000105329)
TGFB2: Transforming growth factor beta 2 (ENSG00000092969)
TIMP3: TIMP metallopeptidase inhibitor 3 (ENSG00000100234)
TNNT2: Troponin T2, cardiac type (ENSG00000118194)
VCL: Vinculin (ENSG00000035403)
VEGFA: Vascular endothelial growth factor A (ENSG00000112715)
Wnt: Wingless-related integration site (composed of 19 Wnt family member genes)
WT: Wild-type
WWTR1: WW domain containing transcription regulator 1 (ENSG00000018408, previous name: TAZ)
YAP1: Yes1 associated transcriptional regulator (ENSG00000137693, previous gene name: YAP)
YY1: YY1 transcription factor (ENSG00000100811, previously also known as Yin Yang 1)
ZMPSTE24: Zinc metallopeptidase STE24 (ENSG00000084073)
